# Computational Evaluation of Improved HIPEC Drug Delivery Kinetics via Bevacizumab-Induced Vascular Normalization

**DOI:** 10.3390/pharmaceutics17020155

**Published:** 2025-01-23

**Authors:** Pouya Namakshenas, Johannes Crezee, Jurriaan B. Tuynman, Pieter J. Tanis, Arlene L. Oei, H. Petra Kok

**Affiliations:** 1 Department of Radiation Oncology, Amsterdam UMC Location University of Amsterdam, 1105 AZ Amsterdam, The Netherlands; h.crezee@amsterdamumc.nl (J.C.); a.l.oei@amsterdamumc.nl (A.L.O.); h.p.kok@amsterdamumc.nl (H.P.K.); 2 Cancer Center Amsterdam, Cancer Biology and Immunology, 1105 AZ Amsterdam, The Netherlands; 3 Cancer Center Amsterdam, Treatment and Quality of Life, 1105 AZ Amsterdam, The Netherlands; j.tuynman@amsterdamumc.nl (J.B.T.); p.tanis@erasmusmc.nl (P.J.T.); 4 Department of Surgery, Amsterdam UMC Location Vrije Universiteit Amsterdam, 1081 HV Amsterdam, The Netherlands; 5 Department of Surgery, Amsterdam UMC Location University of Amsterdam, 1105 AZ Amsterdam, The Netherlands; 6 Department of Surgical Oncology and Gastrointestinal Surgery, Erasmus MC Cancer Institute, 3015 GD Rotterdam, The Netherlands; 7 Laboratory for Experimental Oncology and Radiobiology (LEXOR), 1105 AZ Amsterdam, The Netherlands

**Keywords:** hyperthermic intrapertioneal chemotherapy (HIPEC), bevacizumab, vascular normalization, computational modeling, peritoneal metastasis

## Abstract

**Background:** Oxaliplatin-based hyperthermic intraperitoneal chemotherapy (HIPEC) using the original 30 min protocol has shown limited benefits in patients with peritoneal metastasis of colorectal cancer (PMCRC), likely due to the short duration, which limits drug penetration into tumor nodules. Bevacizumab, an antiangiogenic antibody that modifies the tumor microenvironment, may improve drug delivery during HIPEC. This in silico study evaluates the availability of oxaliplatin within tumor nodules when HIPEC is performed after bevacizumab treatment. **Methods:** Using a computational fluid dynamics (CFD) model of HIPEC, the temperature and oxaliplatin distribution within the rat abdomen were calculated, followed by a model of drug transport within tumor nodules located at various sites in the peritoneum. The vascular normalization effect of the bevacizumab treatment was incorporated by adjusting the biophysical parameters of the tumor nodules. The effective penetration depth values, including the thermal enhancement ratio of cytotoxicity, were then compared between HIPEC alone and HIPEC combined with the bevacizumab treatment. **Results:** After bevacizumab treatments at doses of 0.5 mg/kg and 5 mg/kg, the oxaliplatin availability increased by up to 20% and 45% when HIPEC was performed during the vascular normalization phase, with the penetration depth increasing by 1.5-fold and 2.3-fold, respectively. Tumors with lower collagen densities and larger vascular pore sizes showed higher oxaliplatin enhancement after the combined treatment. Bevacizumab also enabled a reduction in the oxaliplatin dose (up to half at 5 mg/kg bevacizumab) while maintaining effective drug levels in the tumor nodules, potentially reducing systemic toxicity. **Conclusions:** These findings suggest that administering oxaliplatin-based HIPEC during bevacizumab-induced vascular normalization could significantly improve drug penetration and enhance treatment efficacy.

## 1. Introduction

Hyperthermic intraperitoneal chemotherapy (HIPEC), following the removal of visible tumor nodules from the peritoneal surface during cytoreductive surgery (CRS), is a potentially beneficial treatment strategy for peritoneal metastasis of colorectal cancer (PMCRC). PMCRC is associated with a poor prognosis, with a median survival of 6 months in the setting of the best supportive care without treatment and 12 months with systemic chemotherapy [1]. CRS in combination with HIPEC was developed as a potential curative intent treatment, thereby addressing the limited effectiveness of systemic chemotherapy in delivering intravenously administered drugs to the peritoneum due to the peritoneal-plasma barrier [2]. The HIPEC approach turns the peritoneal-plasma barrier into an advantage by utilizing direct locoregional delivery of chemotherapeutics into the peritoneum. This method achieves much higher drug concentrations in the peritoneum compared to the plasma while also leveraging the synergistic effects of heat (41–43 °C) on tumor cell death, as confirmed by both preclinical and clinical studies [3,4,5,6]. Mitomycin C and oxaliplatin are commonly used drugs administered during HIPEC for the treatment of PMCRC, with oxaliplatin showing enhanced cytotoxicity when combined with heat [3]. Although oxaliplatin-based HIPEC following CRS has extended the median survival to 40–50 months [7,8], the recent PRODIGE 7 randomized clinical trial showed no additional benefit of a short 30 min HIPEC protocol with 360–460 mg/m^2^ of oxaliplatin (dosage based on the patient’s body surface area) in combination with CRS compared with CRS alone [8]. In addition, this HIPEC protocol has been used for preventing peritoneal metastasis in the PROPHYLOCHIP and COLOPEC trials, both of them revealing no significant impact on oncological outcomes [9,10].

The efficacy of the chemotherapeutic agents used in HIPEC for targeting residual cancer cells from PMCRC is strongly affected by the colorectal cancer consensus molecular subtypes (CMSs), with a dominant presence of the more treatment-resistant subtypes in PMCRC. However, tumors often exhibit heterogeneous and abnormal vasculature within their microenvironment. This poses two major challenges for intraperitoneal drug delivery. First, the immature neovasculature, characterized by an abnormal basement membrane and reduced pericyte coverage of endothelial cells, is often hyperpermeable. This hyperpermeability causes high interstitial fluid pressure (IFP), creating a strong outward flux that limits drug penetration into the tumor core. Secondly, the hyperpermeable vasculature facilitates the washout of drug molecules that have already entered the tumor interstitium. The imaging of the platinum distribution in the tumor shows that platinum accumulates in the outer border region of the tumor nodule, while the concentrations remain low in the inner region [2,11,12]. A recent study [13] highlighted that the platinum distribution in ovarian cancer tumors has a significant impact on patient survival. Using LA-ICP-MS imaging, the researchers examined the platinum distribution at the tumor–stroma boundary. The study found that those patients with markedly lower platinum levels in the tumor tissue compared to the stroma had worse survival outcomes, whereas the patients with comparable platinum levels in both the stroma and tumor tissue had better survival outcomes. A potential strategy to enhance drug accumulation in tumor nodules is to modify the tumor microenvironment by administering antiangiogenic agents such as bevacizumab, which is FDA-approved for metastatic colorectal cancer [14].

Bevacizumab is a monoclonal antibody that blocks the vascular endothelial growth factor (VEGF), which can normalize the structure and function of the tumor vasculature when administered at the appropriate dose and schedule [15,16]. This vascular normalization prunes abnormal and immature tumor neovasculature while promoting vessel maturation by stabilizing the basement membrane and increasing the pericyte coverage around the vessels [15,17,18]. As a result, tumor microvascular density (MVD) and permeability are reduced [15,19,20]. Consequently, applying intraperitoneal chemotherapy (IPEC) after vascular normalization lowers the likelihood of unwanted drug washout through the vasculature, a critical factor in ensuring effective drug penetration and accumulation in tumor lesions [21]. There is also strong evidence that antiangiogenic treatment reduces the abnormally high IFP in tumors, which is considered to be a barrier to the transport of drug molecules within the tumor [16,22]. While the pharmacokinetic benefits of intravenous chemotherapy combined with bevacizumab have been well studied [16] and shown to provide a favorable benefit--risk profile in metastatic colorectal cancer [23,24], its impact on IPEC in colorectal cancer with peritoneal carcinomatosis remains underexplored. The registered phase II BEV-IP trial [25], the results of which are yet to be published, aims to evaluate the efficacy of perioperative chemotherapy with bevacizumab in PMCRC patients who are eligible for CRS and oxaliplatin-based IPEC.

Gremonprez et al. [26] conducted a study to examine how bevacizumab affects the pharmacokinetics of oxaliplatin-based IPEC in a mouse model of peritoneal CRC xenografts. They observed a 25% reduction in tumor MVD and a significant reduction in the mean IFP from 2.93 kPa to 0.5 kPa. The platinum-to-phosphorus intensity in the outer ring of the tumor, an indicator of oxaliplatin concentration, was approximately two times higher in tumors with lower IFP. However, the oxaliplatin concentration remained very low in the central regions of the tumor. The large tumor nodules in this study, with an average volume of approximately 125 mm^3^, are likely to have hypoxic central regions that impede drug penetration. From an in silico fluid mechanics perspective, the rate of fluid filtration across the vessel wall relative to that across the interstitium represented by the nondimensional parameter α was found by Jain et al. [27] to be most responsive to antiangiogenic treatment when α falls within the range of 1 to 6. The α parameter is a function of tumor size, MVD, and the hydraulic conductivity of the vessel wall and interstitium. The residual tumor after CRS is typically of a microscopic size (R1) and at least less than 2.5 mm in size (R2a). Given a tumor diameter of 2.5 mm and an MVD range for mammary carcinoma of 120 to 260 cm^2^/cm^3^ [28], the α for untreated tumors falls within 3.5 to 5.5. This range indicates the optimal balance for vascular normalization: higher values (α>6) correspond to excessive fluid retention and saturated IFP that remains poorly responsive to changes in α following vascular normalization, whereas lower values (α<1) result in insufficient fluid convection within the tumor, with changes in α having a minimal impact on mitigating IFP. This suggests that tumors classified as R2a are suitable candidates for neoadjuvant antiangiogenic treatment based on their size.

A dedicated computational platform for HIPEC is highly beneficial for evaluating the efficacy of various HIPEC configurations and testing novel hypotheses, potentially leading to optimized treatment approaches. Since 2020, our center has been developing treatment planning software for HIPEC using OpenFOAM v9, an open-source computational fluid dynamics (CFD) software package. This software has been validated by comparing its estimated temperature ranges and drug concentrations with experimental measurements from both rat and human models [29,30,31,32]. Our HIPEC software can estimate the spatiotemporal distribution of temperature and drug concentrations in the parietal and visceral peritoneum, key sites of peritoneal metastasis, as well as their dynamic profiles in the systemic compartment. These data are critical for optimizing treatments by focusing on higher concentrations in targeted regions while controlling the systemic dose and temperature to avoid toxicity. Given the key information we can obtain from HIPEC simulations using our treatment planning tools, we can investigate the effect of neoadjuvant antiangiogenic treatment on the pharmacokinetics of oxaliplatin, aiming to enhance its concentration within the tumor while carefully regulating its levels in the systemic compartment.

**Figure 1:**
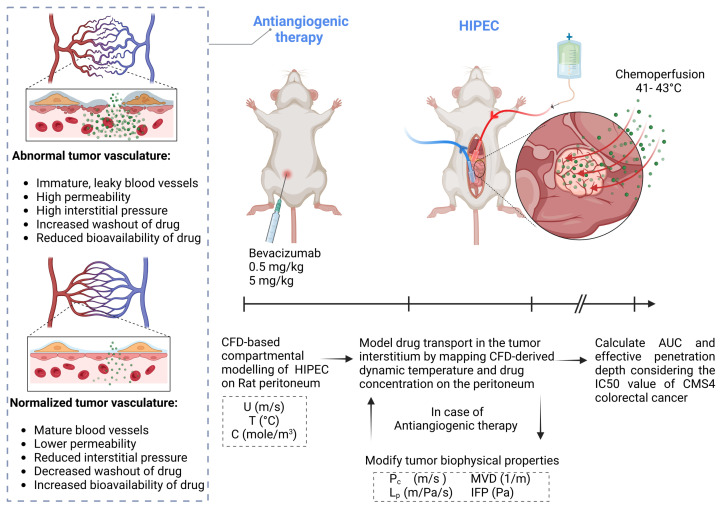
Schematic overview of the present study, investigating the effect of bevacizumab treatments at doses of 0.5 mg/kg and 5 mg/kg on oxaliplatin concentration in peritoneal tumor nodules during HIPEC. CFD simulations were performed to solve for perfusate velocity (U), temperature (T), and oxaliplatin concentration (C), followed by detailed modeling of drug penetration incorporating the altered tumor biophysical properties resulting from bevacizumab treatment. The model incorporates the vascular normalization effect of bevacizumab, resulting in reduced microvessel density (MVD), hydraulic conductivity ($L_{p}$), interstitial fluid pressure (IFP), and vascular permeability ($P_{c}$), which collectively decrease drug washout. Oxaliplatin accumulation was assessed by calculating the area under the curve (AUC) of the concentration–time profile, and the effective penetration depth was determined using IC50 value for CMS4 colorectal cancer. This figure was created via BioRender.com.

In this study, through numerical simulations, we explored the potential of combining HIPEC with an antiangiogenic treatment using bevacizumab to enhance the oxaliplatin penetration into tumor nodules situated on the peritoneal and retroperitoneal surfaces during a 30 min HIPEC procedure (Figure 1). We also investigated how tumor-specific factors, such as collagen fiber density and vascular pore size, influence responsiveness to the combined treatment. Finally, we assessed the potential of this strategy to manage systemic oxaliplatin-related toxicity by comparing the tumor penetration levels and systemic exposure across various oxaliplatin doses. Notably, we focused on the CMS4 subtype of colorectal cancer, known for its enrichment in PMCRC, to evaluate its specific response to this treatment approach.

## 2. Materials and Methods

In this section, we describe the methodology used to investigate the pharmacokinetics of oxaliplatin during HIPEC in combination with the vascular normalization effects of bevacizumab. First, we explain the geometry and solution strategy. Subsequently, we describe the mathematical equations. Next, we detail how the effect of bevacizumab treatment was modeled. Then, we describe how we assess the association of extracellular matrix stiffness and microvasculature permeability with the improved efficacy of the combined treatment. Finally, we describe the data evaluation process and the metrics used for data analysis.

### 2.1. Solution Strategy and Model Geometry

Due to the different size scales between the abdomen and the tumor nodule, two separate simulations were performed: one to calculate the distribution of heated chemoperfusion by the bulk motion of fluid and the other to model the transport of oxaliplatin in the tumor nodule. Specifically, the temporal drug concentration on the peritoneal and retroperitoneal surfaces was used as the boundary condition for the drug penetration analysis. In this section, we explain the geometry and case setups for both analyses: CFD-driven oxaliplatin transport in the abdomen and its penetration into the tumor nodules.

The computational geometry for CFD analysis of HIPEC is a 3D model of a rat abdomen, replicating an open-abdomen procedure and including intra-abdominal organs, similar to the geometry used in previous studies [30,33]. The simulation was conducted using the finite volume method for solving governing equations. The rat model was used because of its lower computational cost for HIPEC simulation, and, although the quantitative results may differ, the underlying concept would remain unchanged if a human geometry model were used. The organs were modeled as solid structures, with the remaining abdominal space allocated for chemofluid perfusion. Given the complexity of the simulations, the model excluded deformation effects caused by flow-related loads on the organs and the abdominal wall. For more details, we refer to [30,33]. The rat model was in supine position, in accordance with positioning during the HIPEC procedure. The inflow was placed toward the back of the peritoneal cavity, while the outflow was located at the fluid surface of the peritoneal cavity. Two different HIPEC setups are considered in this study to account for the variability in drug distribution that may result from catheter positioning. These setups are distinguished by the position of the outflow. In the first setup, the inflow is positioned between the liver and the intestine, while the outflow is placed at the maximum transverse distance from the inflow, above the intestine. In the second setup, the inflow placement remains the same, but the outflow is positioned further away at the maximum longitudinal distance from the inflow, near the rectum. The fluid flow was modeled as laminar due to a Reynolds number of approximately 600. The geometry mesh is composed of 66,000 hexahedral cells.

For drug penetration analysis, a peritoneal tumor nodule with a diameter of d=2mm, consistent with the R2a scoring of CRS, was modeled such that half of the tumor was embedded in the host tissue while the other half was exposed to chemoperfusion [34]. The average temperature values induced on the organ’s surface were used to adjust the sink term in the drug transport equation for the tumor to account for the increased drug accumulation due to the temperature rise. The element size for both the tumor and the host tissue (which is five times the size of tumor) was refined using a mesh size of 0.05 mm to capture the penetration depth of the drug.

### 2.2. Initial and Boundary Conditions

The initial temperature of the rat abdomen was set to the normal body temperature of 37 °C, with an initial drug concentration of zero and an initial pressure equal to atmospheric pressure. Four boundary conditions were applied in the HIPEC model: (1) a constant inflow rate of 60 mL/min at 43 °C with an equivalent outflow, combined with a chemoperfusate concentration of 160 μM and a zero-gradient condition at the outflow; (2) a no-slip velocity condition at the fluid–tissue interface, ensuring continuity and smooth diffusion of temperature and drug concentration gradients; (3) heat loss from the open abdomen to the environment, modeled as a 600 W/m^2^ flux, accounting for evaporation, convection, and radiation; and (4) additional heat loss through the peritoneal surface to the environment, incorporating thermal resistance effects.

In the explicit model of drug transport from the peritoneal cavity to tumor nodules, the CFD-derived, surface-averaged oxaliplatin concentration for each abdominal organ was applied as the boundary condition to the exposed surface tumor nodule during chemoperfusion. The boundary conditions used to model chemotherapeutic delivery in HIPEC are listed in Table 1. For further details, we refer to our previous work [33].

### 2.3. Governing Equations

The fundamental physics of the CFD analysis for HIPEC involves fluid flow, heat transfer, and mass transport. Within the peritoneal cavity, which is treated as a fluid domain, we solve the continuity and momentum equations for incompressible flow, together with the energy and passive scalar drug transport equations, as illustrated by Equations (1)–(4).

(1) $$\nabla \cdot \vec{U}=0,$$

(2) $$\frac{\partial \vec{U}}{\partial t}+\nabla \cdot \left(\vec{U}\times \vec{U}\right)-\nabla \cdot \left(\nu \nabla \vec{U}\right)=-\frac{1}{\rho }\nabla p+\rho \vec{g},$$

(3) $$\frac{\partial \rho h}{\partial t}+\nabla \cdot \left(\rho \vec{U}h\right)+\frac{\partial \rho K}{\partial t}+\nabla \cdot \left(\rho \vec{U}K\right)-\frac{\partial p}{\partial t}=-\nabla \cdot \vec{q},$$

(4) $$\frac{\partial C}{\partial t}+\nabla \cdot \left(\vec{U}C\right)-\nabla \cdot \left(D\nabla C\right)=0,$$

In Equations (1) and (2), the variables $\rho ,\vec{U},\nu ,p,\vec{g}$ represent the density $\left[{\mathrm{kg}/m}^{3}\right]$, flow velocity [m/s], kinematic viscosity $\left[m^{2}/s\right]$, pressure [Pa], and the gravitational vector $\left[{m/s}^{2}\right]$, respectively. In Equation (3), the variables $h,K,\vec{q}$ denote the specific enthalpy (enthalpy per unit mass) $\left[m^{2}/s^{2}\right]$, specific kinetic energy ($K={\left|U\right|}^{2}/2$) $\left[m^{2}/s^{2}\right]$, and heat flux $\left[{W/m}^{2}\right]$. In Equation (4), *C* represents the drug concentration $\left[{\mathrm{mol}/m}^{3}\right]$ and *D* is the diffusion coefficient of drug molecule $\left[m^{2}/s\right]$.

For each of the abdominal organs, modeled as distinct solid domains, we solve the Pennes bioheat equation together with the drug transport equation, represented by Equations (5) and (6), respectively.

(5) $$\rho_{t}c_{t}\frac{\partial T}{\partial t}-k_{t}\nabla^{2}\left(T\right)=-Q_{therm},$$

(6) $$\frac{\partial C}{\partial t}-\nabla \cdot \left(D\nabla C\right)=-S_{c},$$

where *T*, *c*, and *k* are the temperature [K], the heat capacity [J/kg/K], and the thermal conductivity [W/m/K], respectively. The subscript *t* indicates tissue-specific parameters. The thermal sink term $Q_{therm}$ represents the heat dissipation $\left[W/m^{3}\right]$ due to blood perfusion in the tissue, which reads as follows:

(7) $$Q_{therm}=\rho_{b}\cdot w_{b}\cdot c_{b}\cdot \left(T_{t}-T_{a}\right),$$

(8) $$\Delta T_{a}=\frac{q_{m}+q_{therm}-q_{skin}-q_{tail}}{\rho_{b}\cdot c_{b}\cdot V_{b}}\cdot \Delta t,$$

where $w_{b},T_{a},q,V_{b},$ and Δt are the blood perfusion rate [$s^{-1}$], the arterial temperature [K], the heat transfer rate [W], the total blood volume [$m^{3}$], and the time step [s], respectively. The subscript *b* indicates blood-specific parameters. As provided in Equation (8), the arterial temperature is updated at each time step based on the balance of heat transfer, which involves heat gain from metabolic activity ($q_{m}$) and tissue perfusion ($q_{therm}$), and heat loss through the rat skin ($q_{skin}$) and tail ($q_{tail}$).

The drug transport model in Equation (6) is primarily diffusion-based and incorporates a sink term $S_{c}$ [$\mathrm{mol}/m^{3}/s$] to account for cellular $S_{cell}$ and vascular absorption $S_{v}$:

(9) $$\begin{array}{cc} S_{c} & =\underset{S_{cell}}{\underset{︸}{\beta \cdot C}}+\underset{S_{v}}{\underset{︸}{\left(-P_{c}\cdot \frac{S}{V}\cdot \frac{Pe_{v}}{e^{Pe_{v}}-1}\right)\cdot C\cdot y\left(T\right)}} \end{array}$$

(10) $$Pe_{v}=\underset{=\mathrm{fluid}\;\mathrm{gain}\;\mathrm{from}\;\mathrm{the}\;\mathrm{vessels}}{\underset{︸}{L_{p}\frac{S}{V}\cdot \left(P_{v}-P_{i}-c\cdot \left(\pi_{v}-\pi_{i}\right)\right)}}\cdot \frac{\left(1-\sigma \right)}{P_{c}\frac{S}{V}},$$

(11) $$\begin{array}{c} y\left(T\right)=-0.04715\times T{(}^{\circ }C)+2.744555 \end{array}$$

where β is the first-order elimination constant [s^−1^], $P_{c}$ is the permeability of the capillary wall for the drug [m/s], and $\frac{S}{V}$ is the blood vessel surface area per unit volume of tissue for transcapillary exchange [m^−1^]. Additionally, $P_{ev}$ denotes the Péclet number [-], $L_{p}$ is the vasculature hydraulic conductivity [m/(Pa · s)], and $P_{v}$ and $P_{i}$ are the vascular pressure and IFP, respectively, both in [Pa]. The vascular and interstitial osmotic pressures, $\pi_{v}$ and $\pi_{i}$, are also measured in [Pa], while σ is the osmotic reflection coefficient [-]. The function y(T) represents an empirical relationship that accounts for the reduction in drug washout to the vasculature under conditions of mild hyperthermia (37–43 °C). [35]. The constants of the normalized linear function y(T) were derived by fitting a curve to the concentration–temperature profile of oxaliplatin, as detailed in [35].

The systemic concentration of oxaliplatin can be calculated by accounting for the vascular uptake of oxaliplatin as a source term and the exchange between the systemic and peripheral compartments.

(12) $$\frac{C_{sys}}{\partial t}=S_{v}-K_{12}\cdot C_{sys}+K_{21}\cdot C_{per}-K_{e}\cdot C_{sys},$$

(13) $$\frac{C_{per}}{\partial t}=K_{12}\cdot C_{sys}-K_{21}\cdot C_{per},$$

where $K_{12}$ and $K_{21}$ represent the rate constants for transfer between the systemic and peripheral compartments, respectively, and $K_{e}$ is the elimination rate constant. For more comprehensive details on the CFD-based compartmental modeling of HIPEC, we refer to [30]. The physical constants for the thermal and drug delivery modules of this study are provided in Table 2 and Table 3, respectively.

### 2.4. Modeling of Vascular Normalization Effect of Bevacizumab Treatment

In this study, we modeled the vascular normalization effect of bevacizumab on tumors at two different doses: 0.5 mg/kg and 5 mg/kg. To this end, we modified the tumor biophysical parameters relevant to Equations (9) and (10) based on observed changes reported in the literature during the normalization window, as shown in the workflow diagram in Figure 1. Bevacizumab at 5 mg/kg resulted in an 80% reduction in the IFP of peritoneal CRC UT29 xenografts [26]. Although we did not find data on changes in IFP at a dose of 0.5 mg/kg in the literature, another study on xenograft carcinoma showed that the IFP of tumors remained unchanged at a dose of 0.2 mg/kg [43]. Therefore, we assume that the IFP level does not change after the administration of a low dose of bevacizumab at 0.5 mg/kg. Since the IFP level is strongly dependent on tumor size, we used an IFP value of 1.4 kPa for untreated tumors with a diameter of 2 mm [34], and we assigned IFP values of 1.4 kPa and 0.25 kPa for tumors treated with 0.5 mg/kg and 5 mg/kg of bevacizumab, respectively. We assumed that the MVD of untreated tumors is 200 1/cm based on the range reported for mammary tumors [28]. The vascular hydraulic conductivity for fluid transport ($L_{p}$) in untreated tumors was established at $2.1\times 10^{-11}$ m/Pa/s [37]. The microvascular permeability for drug transport ($P_{c}$) in untreated tumors was taken from the reported value for sucrose in the mesentery, which is equal to $1.43\times 10^{-6}$ m/s [28]. The molecular weight of sucrose is close to that of oxaliplatin.

In xenograft tumors derived from human colorectal cancer cells (LS174T), the MVD was reduced by 45% and 65% with bevacizumab treatments at doses of 0.5 mg/kg and 5 mg/kg, respectively [19]. Vascular leakage in that study [19] remained unchanged at the 0.5 mg/kg dose but was reduced by 22% at the 5 mg/kg dose. We assumed that the hydraulic conductivity and permeability of the vasculature would follow a similar pattern of change at these doses. Reductions of 35% and 50.3% in vascular permeability were also reported in another study [44] for low-VEGF-secreting and high-VEGF-secreting melanoma xenografts, respectively, following treatment with 5 mg/kg of bevacizumab.

### 2.5. Modeling the Impact of Tumor Stiffness and Microvascular Permeability

The stiffness of the tumor’s extracellular matrix significantly affects the transport of therapeutic agents within the tumor. Increased stiffness hinders interstitial fluid flow and impedes the penetration of therapeutic agents into the central region of the tumor [45].

The stiffness of a tumor is closely linked to the density and organization of collagen fibers in the tumor’s extracellular matrix [46,47]. Hydraulic conductivity—defined as the ease with which interstitial fluid flows through the interstitium–shows a strong inverse correlation with collagen fiber density in peritoneal metastasis [48]. The impact of tumor stiffness on transport hindrance is particularly significant for large molecules, although small molecules also experience notable hindrance in tumors with high variability in fiber density.

We modeled the influence of tumor stiffness on the response to vascular normalization by bevacizumab and HIPEC, implicitly adjusting the effective diffusion coefficient of oxaliplatin. The fiber density for PMCRC ranges between 4% and 32% [48], which is associated with a reduction in the effective diffusion coefficient by a factor of ≈0.5 [49]. Additionally, denser tumors exhibit reduced microvascular growth [50]. Therefore, we set the MVD of tumors with low collagen fiber content to twice that of those with high collagen fiber content. Microvascular permeability levels can significantly influence drug washout during HIPEC. We evaluated how the sensitivity of microvascular permeability affects the pharmacokinetics of oxaliplatin when HIPEC is combined with bevacizumab treatment. We modeled permeability values at 0.2, 0.5, 1, 2, and 5 times the reference $P_{c}$, which was set to $1.43\times 10^{-6}$ m/s. Given that the reduction in $P_{c}$ was reported to be substantial for the 5 mg/kg bevacizumab dose but not for the 0.5 mg/kg dose, as discussed in the previous subsection, we excluded the 0.5 mg/kg bevacizumab treatment from this analysis.

### 2.6. Evaluation Metrics and Data Analysis

To compare drug accumulation in the tumor, we first analyze the evolution of the average volumetric drug concentration in the tumor and the AUC of that profile, as shown in Equations (14) and (15), respectively. Equation (14) represents the spatial average at a specific time point, while Equation (15) integrates over time to assess the total drug exposure through AUC calculation.

(14) $$\begin{array}{c} \bar{C_{t}}=\frac{1}{V_{t}}\int_{V_{t}}C_{t}\left(x,y,z\right)\;dV \end{array}$$

(15) $$\begin{array}{c} \mathrm{AUC}=\int_{0}^{t_{f}}\bar{C_{t}}\left(t\right)\;dt \end{array}$$

where $V_{t}$ is the tumor volume and $t_{f}$ is the duration of the HIPEC treatment. Next, the effective drug penetration depth ($d_{\mathrm{eff}}$) is defined as the depth of the tumor exposed to a dose of oxaliplatin exceeding 10% of the oxaliplatin IC50, measured in short-term cell viability assays at the given temperature during HIPEC. Hyperthermia enhances the cytotoxicity of oxaliplatin, resulting in a reduction in its IC50. We define the thermal enhancement ratio (TER) as the ratio of the IC50 at normothermic temperature to the IC50 at hyperthermic temperature. The mean oxaliplatin IC50 and TER for the CMS4 subtype of colorectal cancer, derived from measurements [3] on the MDST8, COLO320, and HUTU80 cell lines, is summarized in Table 4. CMS4 is the most prevalent subtype in PMCRC [51].

To evaluate how bevacizumab treatment can contribute to the reduction in the administered dose of oxaliplatin, several simulations were conducted by increasing the base dose of 160 µM in increments of 20 µM up to 320 µM. The systemic concentration of the administered dose that can provide comparable effective penetration to 160 µM when combined with the vascular normalization effects of 0.5 mg/kg and 5 mg/kg of bevacizumab was then compared. We define the pharmacokinetic advantage (PA) ratio as the ratio of the AUC of drug concentration in the tumor nodule to the AUC in the systemic compartment (plasma):

(16) $$\begin{array}{c} \mathrm{PA}=\frac{{\mathrm{AUC}}_{\mathrm{tumor}}}{{\mathrm{AUC}}_{\mathrm{plasma}}} \end{array}$$

## 3. Results

The results section includes a subsection on the simulated oxaliplatin distribution and temperature driven by fluid motion during HIPEC. This is followed by a subsection illustrating the availability of oxaliplatin in PMCRC tumor nodules through effective penetration depth and AUC. Next, a subsection presents the results related to the responsiveness of tumor nodules to the combined treatment based on their stiffness and microvascular permeability. Finally, a subsection discusses the potential of bevacizumab treatment in managing the systemic toxicity associated with oxaliplatin.

### 3.1. CFD-Derived Drug and Temperature Distribution

**Figure 2:**
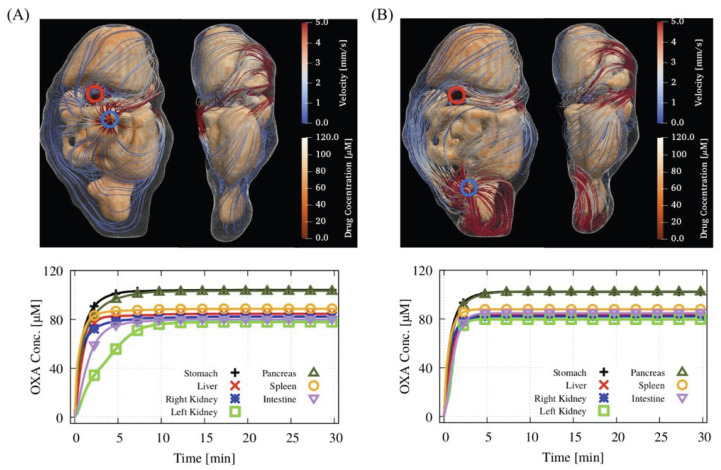
Distribution of oxaliplatin on the surfaces of abdominal organs due to bulk fluid flow. The data were derived from (**A**) the first catheter setup, where inflow (indicated by a red circle) and outflow (indicated by a blue circle) occur at maximal transverse distance, and (**B**) the second catheter setup, where inflow and outflow occur at maximal longitudinal distance. The 3D panels present the velocity contours alongside the oxaliplatin concentration on the surfaces of abdominal organs at the end of HIPEC. The line graphs represent the surface-averaged oxaliplatin concentration throughout the 30 min HIPEC chemoperfusion.

The coverage of oxaliplatin during HIPEC on the surfaces of abdominal organs, potential sites for peritoneal tumor nodules, was derived from CFD simulations based on catheter setups one and two, as illustrated in Figure 2A,B, respectively. The 3D plot panels display the velocity contours along with the surface distribution of oxaliplatin at the end of the 30 min HIPEC. The line graphs illustrate the surface-averaged evolution of oxaliplatin on the surfaces of each organ during HIPEC. The data indicate that setup 2, where the inflow and outflow have the maximal longitudinal distance, achieves faster coverage of oxaliplatin, particularly on the left kidney and intestine. The steady-state levels of oxaliplatin concentration from both setups are comparable. Table 5 presents the steady-state temperature levels achieved during HIPEC using both catheter setups, as well as the IC50 of oxaliplatin at these temperatures on the peritoneal and retroperitoneal surfaces surrounding the abdominal organs. There is less heterogeneity in the second setup. The temperature and IC50 range from 38.4 to 41.7 °C and from 338 µM to 234 µM, respectively, for the first setup and from 40.5 to 41.9 °C and from 278 µM to 231 µM for the second setup.

### 3.2. Comparison of Oxaliplatin Accumulation and Penetration Depth

**Figure 3:**
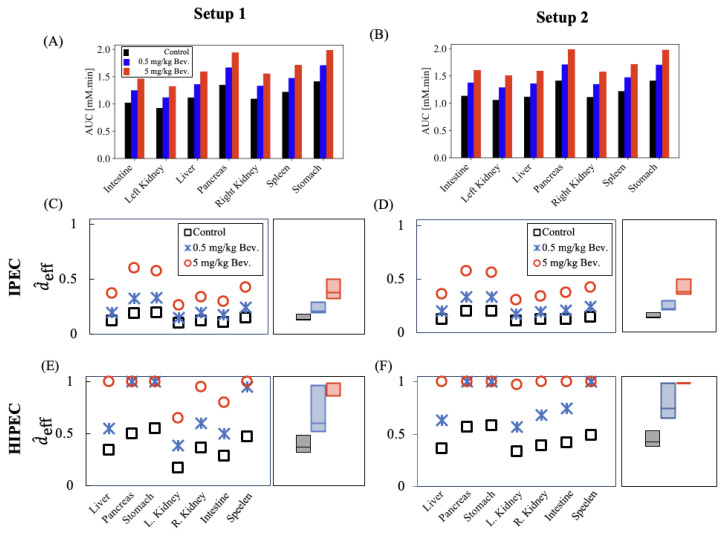
Comparison of bevacizumab’s vascular normalization effects on oxaliplatin availability in tumor nodules, assessed by AUC and effective penetration depth values based on IC50 at specific temperatures using catheter setups one (**A**,**C**,**E**) and two (**B**,**D**,**F**). (**A**,**B**) AUC of oxaliplatin in tumor nodules at peritoneal and retroperitoneal surfaces. (**C**,**D**) Normalized effective penetration depth (${\hat{d}}_{\mathrm{eff}}$) values for IPEC and (**E**,**F**) for HIPEC, with box plots showing interquartile penetration depth values.

Figure 3A,B show the AUC values of volumetric-averaged oxaliplatin concentration in tumor nodules located at the peritoneal and retroperitoneal surfaces of various organs using the two catheter setups, respectively. These panels compare the AUC levels at the end of HIPEC under control conditions and with the vascular normalization effect of bevacizumab at doses of 0.5 mg/kg and 5 mg/kg. The AUC increased by up to 20% and 45% with lower and higher doses of bevacizumab, respectively. Figure 3C,E (for catheter setup one) and Figure 3D,F (for catheter setup two) illustrate the normalized effective penetration depth values without the heat effect (IPEC) and with the heat effect (HIPEC), respectively. The box plot provides an overall evaluation, displaying the interquartile range of the penetration depth values across different tumor locations. The effective penetration of HIPEC is greater than that of IPEC as the cytotoxicity of oxaliplatin is enhanced by the TER ratio. The median penetration depth increases by 1.5-fold and 2.3-fold, respectively, when HIPEC is combined with the vascular normalization effect of bevacizumab at doses of 0.5 mg/kg and 5 mg/kg.

### 3.3. The Effect of Tumor Stiffness and Microvascular Permeability on Oxaliplatin Availability

**Figure 4:**
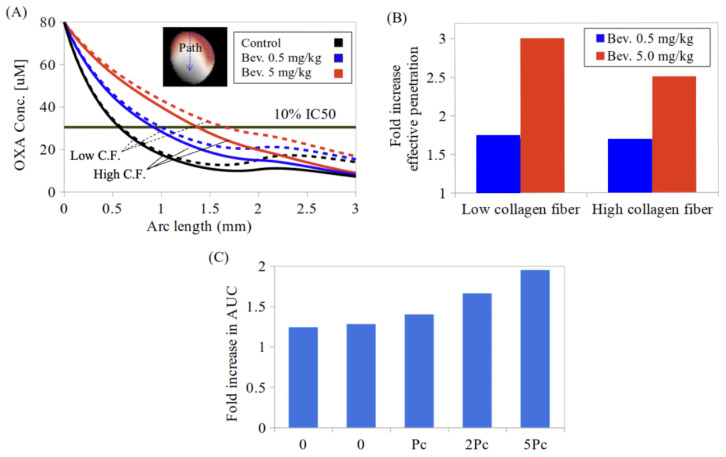
Simulated effect of (**A**,**B**) collagen fiber density (4% and 32%) and (**C**) microvascular permeability (ranging from $0.2\;P_{c}$ to $5\;P_{c}$) on the availability of oxaliplatin in the tumor interstitium following bevacizumab treatment at two doses (0.5 and 5 mg/kg) and HIPEC. The control case is HIPEC alone. (**A**) Oxaliplatin concentration profile from the tumor surface to a depth of 3 mm. The region from 0 to 2 mm represents the tumor, while 2 to 3 mm corresponds to normal tissue. Solid lines represent tumors with high collagen fiber (CF) density, and dashed lines represent tumors with low collagen fiber density. The IC50 value corresponds to a temperature of 40.1 °C. (**B**) Fold increase in effective penetration depth compared to the control case. (**C**) Fold increase in the AUC of oxaliplatin compared to the control case at various permeability levels.

Figure 4A illustrates the penetration depth values of oxaliplatin in PMCRC tumors with low (4%) and high (32%) collagen fiber density. The comparisons include HIPEC alone and the combination therapy of bevacizumab-induced vascular normalization at two doses (0.5 and 5 mg/kg) with HIPEC. The fold increase in the effective penetration depth of tumor nodules with low- and high-collagen fiber density, compared to the control case (without bevacizumab treatment), is shown in Figure 4B. The tumor is located on the peritoneal surface of the intestine, with a temperature of 40.1 °C and an IC50 of 305 µM (see Table 5). The arc length starts from the tumor surface, which is directly exposed to the chemoperfusate and extends up to 3 mm, with 1 mm representing the normal tissue region. Those tumors with lower fiber density exhibited higher oxaliplatin concentrations along the path. These predicted results demonstrate that tumors with lower fiber density benefit more from the combination treatment with 5 mg/kg of bevacizumab. However, at 0.5 mg/kg, the results are comparable between both fiber densities.

The effect of microvascular permeability following the combined treatment on the fold increase in oxaliplatin AUC is illustrated in Figure 4C. The AUC of oxaliplatin increases more significantly at higher permeability levels. For permeability levels ranging from $0.2\;P_{c}$ to $5\;P_{c}$, the fold increases in AUC are 1.25 and 1.95, respectively.

### 3.4. Potential of Bevacizumab to Reduce Oxaliplatin Systemic Toxicity

**Figure 5:**
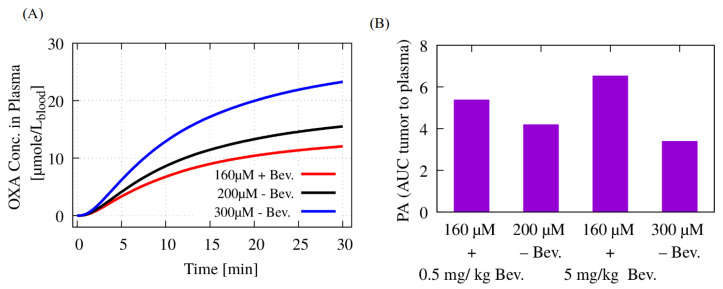
(**A**) Time course of oxaliplatin concentration in plasma during 30 min HIPEC at different doses (160 μM with bevacizumab; 200 μM and 300 μM without bevacizumab). (**B**) Pharmacokinetic advantage (PA) ratio comparison between combination therapy (160 μM oxaliplatin + bevacizumab) and HIPEC monotherapy (200 μM and 300 μM oxaliplatin). The combination of 160 μM oxaliplatin-based HIPEC with bevacizumab (0.5 mg/kg and 5 mg/kg) is predicted to provide a greater pharmacokinetic advantage compared to higher doses of oxaliplatin in HIPEC alone.

The simulation results demonstrate the combination of a base oxaliplatin dose of 160 μM during HIPEC following bevacizumab treatment (0.5 mg/kg or 5 mg/kg) achieved drug penetration levels comparable to higher oxaliplatin HIPEC doses alone (200 μM or 300 μM, respectively), as shown in Table 6. This enhanced penetration is attributed to the incorporation of the vascular normalizing effects of bevacizumab in the model. The evolution of the oxaliplatin concentration in the systemic compartment (plasma) and its associated PA ratio, the AUC of oxaliplatin in the tumor to plasma, for each strategy are depicted in Figure 5A,B, respectively. At the end of the 30 min HIPEC, the oxaliplatin concentration increases from $12\;\;\mu {\mathrm{mol}/L}_{\mathrm{blood}}$ to 15 $\mu {\mathrm{mol}/L}_{\mathrm{blood}}$ and 24 $\mu {\mathrm{mol}/L}_{\mathrm{blood}}$ for the dose increase from 160 μM to 200 μM and 300 μM, respectively. Compared to HIPEC monotherapy using 200 μM and 300 μM oxaliplatin, the combination treatment (160 μM oxaliplatin + bevacizumab) achieved approximately 28% and 92% higher PA values, respectively.

## 4. Discussion

In the present study, we conducted a numerical investigation of the potential of bevacizumab in the neoadjuvant regimen for the treatment of PMCRC prior to oxaliplatin-based HIPEC. Our results suggest that the combination of bevacizumab with HIPEC can significantly enhance oxaliplatin accumulation in R2a-score residual tumor nodules after CRS compared to HIPEC alone. We have also demonstrated that, in the combined treatment, when hyperthermia is applied during intraperitoneal chemotherapy (i.e., HIPEC), the effective drug penetration is considerably higher than at normothermic temperatures (i.e., IPEC). According to our predictions, PMCRC tumor nodules with lower collagen fiber density and higher permeability respond more effectively to the combined HIPEC and bevacizumab treatment. Furthermore, we demonstrated that combining HIPEC with the bevacizumab treatment enables comparable oxaliplatin penetration in the tumor at a lower administered dose, thereby managing the oxaliplatin accumulation in the systemic compartment more effectively than HIPEC alone.

The potential benefits of bevacizumab in the neoadjuvant setting for PMCRC patients undergoing IPEC, particularly in terms of pharmacokinetic effects such as drug penetration and availability in tumor nodules, remain inadequately explored in the literature. Using numerical simulations, we showed that bevacizumab-induced vascular normalization enhances the intraperitoneal drug delivery kinetics by reducing the tumor microvascular density and normalizing the vascular permeability. These effects reduce the likelihood of intraperitoneally administered chemotherapy agents being washed out of the tumor lesions into systemic circulation. This stabilization helps to maintain a high concentration gradient of the chemotherapeutic agent within the peritoneal cavity, particularly in those tumor regions that are directly exposed to the agent. As a result, the drug penetration into tumor lesions is enhanced. Our results (Figure 3) further highlight that the effective drug penetration into tumor nodules was significantly enhanced when HIPEC was administered during the time window of tumor microenvironment alteration following the bevacizumab treatment. Specifically, when HIPEC was combined with the bevacizumab treatment at a dose of 5 mg/kg, the variability in the penetration depth within the tumor nodules across the peritoneum was reduced. This approach could potentially lead to improved treatment outcomes for patients. An observational study [52] of 61 participants receiving neoadjuvant systemic chemotherapy found that the addition of bevacizumab to the chemotherapy regimen for PMCRC patients undergoing CRS and HIPEC significantly improved the median overall survival, extending it from 22 months to 39 months. A multivariate analysis showed a hazard ratio of 0.31 (*p* = 0.019) for the group receiving neoadjuvant chemotherapy with bevacizumab. In another prospective cohort study [53] involving 97 patients, lower levels of intraperitoneal VEGF measured immediately after the surgical incision were associated with improved survival in patients with peritoneal metastasis treated with CRS and HIPEC. The study highlighted bevacizumab’s potential in controlling both intravenous and intraperitoneal VEGF levels, which may lead to better treatment outcomes.

There is a close correlation between the dose of oxaliplatin administered during HIPEC and its concentration in the tumor. In a phase I clinical trial, Elias et al. [54] investigated the pharmacokinetics of oxaliplatin-based HIPEC conducted via an open-abdomen approach. The HIPEC protocol described in [54] involved administering fixed doses of oxaliplatin to different patients with peritoneal carcinomatosis, with the doses ranging from 260 to 460 mg/m^2^, in increments of 50 mg/m^2^. Each dose was administered at a flow rate of 2 L/min and a target intraperitoneal temperature of 42–44 °C over a 30 min period. The tumor exposure to oxaliplatin increased by 1.5-fold when comparing the lowest dose of 260 mg/m^2^ to the highest dose of 460 mg/m^2^. Stewart et al. [55], in a phase I clinical trial using a different oxaliplatin-based HIPEC protocol, evaluated two doses of oxaliplatin—200 mg/m^2^ and 250 mg/m^2^. This protocol involved a 120 min perfusion at a flow rate of 0.8 to 1 L/min with a target outflow temperature of 40 °C. The study found that the tumor oxaliplatin levels were 1.3 times higher with the 250 mg/m^2^ dose compared to the 200 mg/m^2^ dose. However, the higher dose was associated with grade 3 toxicity lasting more than 5 days, reaching dose-limiting toxicity. Despite the potential for enhancing the effectiveness of HIPEC by increasing tumor exposure to oxaliplatin through higher intraperitoneal concentrations, a Dutch multicenter study [56] found no significant correlation between the variability in the oxaliplatin concentrations (91.9 to 377.6 mg/L) and survival benefits in PMCRC patients. From a pharmacokinetic perspective, the efficacy of IPEC and HIPEC should be determined by the drug’s ability to penetrate the tumor such that its concentration exceeds the inhibitory concentration rather than just by overall enhanced accumulation. This is because there could be bias if the drug accumulates only in a local region as this would merely raise the concentration above the threshold needed for cancer cell killing without ensuring effective treatment throughout the tumor. Therefore, we used the IC50 value of oxaliplatin for the most prominent subtype, CMS4, in PMCRC as a threshold to determine the effective penetration depth (Table 4 and Figure 3).

In drug delivery for small-molecular-weight agents such as oxaliplatin, concentration-gradient-driven diffusion is the primary transport mechanism. Accordingly, we evaluated the oxaliplatin penetration in the combined treatment setting based on the parameters that influence diffusion. Variability in tumor microstructural properties, such as tortuosity and porosity, has less of an effect on drug transport for small molecules than it does for larger agents [57]. However, the diffusion hindrance still varies up to twofold across the range of collagen fiber densities reported for PMCRC tumor nodules as collagen density is correlated with porosity. Our findings suggest that tumors with lower collagen fiber density are more responsive to a combined bevacizumab and HIPEC treatment (Figure 4). The pore size within the tumor vasculature presents another variable that affects drug washout during HIPEC, which is also diffusion-driven. During vascular normalization, the pore size in tumor vasculature tends to decrease, thereby reducing permeability. For instance, a reduction in the pore size from 400 nm to 100 nm was observed in E0771 tumors following the administration of 5 mg/kg DC101 [58], an anti-VEGFR-2 antibody (rat anti-mouse). According to our results, the combined treatment is more effective in targeting tumors with larger pore sizes (Figure 4). In light of these findings, further studies should determine whether there is a significant relationship between the clinicopathological features of tumors and factors such as collagen fiber density and pore size. This approach could help to identify the most responsive tumor types, enabling prioritized selection of patients for the combined treatment.

Oxaliplatin-based regimens are often associated with hematologic toxicities, including neutropenia (abnormally low neutrophil count) and thrombocytopenia (abnormally low platelet count). In this study, we demonstrated that combining HIPEC with a 5 mg/kg dose of neoadjuvant bevacizumab results in oxaliplatin tumor penetration comparable to that achieved by nearly doubling the oxaliplatin dose during HIPEC (Figure 5). This finding suggests that, in high-risk populations, particularly those with low platelet levels, the dose of oxaliplatin could be safely reduced by incorporating bevacizumab into the neoadjuvant regimen. Additionally, some studies have shown that adding bevacizumab to oxaliplatin-based chemotherapy of metastatic colorectal cancer provides significant protective effects against oxaliplatin-induced complications. For example, Overman et al. [59] reported reduced thrombocytopenia rates (19% vs. 51%, *p* < 0.001), and Zhou et al. [60] observed a similar effect (31.7% vs. 77.2%, *p* = 0.02).

Despite uncertainties in the baseline parameters and the heterogeneity of the tumor microstructure that may quantitatively influence the models, the simulation results fall within a reasonable range compared to the reported experimental measurements, such as the dose in the systemic compartment [11] and oxaliplatin penetration into the tumor nodules [61]. Consistent with the experimental observations [26], our simulation results (Figure 4) show that the normal tissue adjacent to the tumor has a higher concentration of oxaliplatin compared to the tumor tissue, particularly in cases where the tumor properties significantly hinder drug transport. Furthermore, according to our results, the accumulation of oxaliplatin was enhanced nearly 1.5 times in the 2 mm tumor nodules with the combined treatment of HIPEC and bevacizumab (Figure 3). Our findings are consistent with those of Gremonprez et al. [26], who performed an in vivo study examining oxaliplatin distribution following IPEC administration during the vascular normalization phase induced by bevacizumab (5 mg/kg) in a mouse model of colorectal carcinomatosis. Their results demonstrated that, within the peritoneal border up to 1.68 mm, the platinum-to-phosphorus intensity increased approximately twofold (*p* = 0.0221, n = 15) with the bevacizumab treatment. Therefore, the findings from the present study not only support the potential of combining HIPEC with bevacizumab for enhanced oxaliplatin delivery but also lay the groundwork for further investigation into optimizing the treatment protocols. The clinical relevance of this approach extends beyond oxaliplatin, particularly in the context of evolving treatment strategies for PMCRC. For example, the INTERACT-PLUS phase II trial [62] introduced a neoadjuvant strategy combining systemic mFOLFOX with bevacizumab and intraperitoneal irinotecan before MMC-based HIPEC. Since oxaliplatin is used only systemically in this protocol, it would be valuable to explore whether the improved penetration mechanisms predicted in this study can also be applied to irinotecan or its active metabolite in the intraperitoneal setting. Future studies should focus on refining the model parameters, particularly by examining tumor heterogeneity—such as variations in the vascular network and architecture, as well as differences in tumor response to bevacizumab. These efforts will require additional in vivo studies to validate the findings in clinical settings.

## 5. Conclusions

This in silico study demonstrates the potential of bevacizumab to enhance oxaliplatin delivery during HIPEC for PMCRC. By accounting for the dose variability with different HIPEC setups and examining the tumor microstructure (collagen fiber density and vascular permeability), our numerical results show that bevacizumab significantly enhances oxaliplatin penetration, particularly in tumors with lower collagen density and higher vascular permeability. In addition, bevacizumab helps to reduce the systemic toxicity associated with oxaliplatin by enabling lower doses of the drug to achieve comparable therapeutic effects. These findings provide valuable insights for optimizing HIPEC treatment protocols and form the basis for future preclinical and clinical studies, including the ongoing BEV-IP phase II trial.

## Figures and Tables

**Table 1:** Boundary conditions for the HIPEC model.

Boundary Type	Mathematical Formulation
1. Flow boundary	$\Gamma_{\mathrm{in}}$: $v=v_{0}$, $T=T_{0}$, $C=C_{0}$
	$\Gamma_{\mathrm{out}}$: ∇T·n=0, ∇C·n=0, $p=p_{\mathrm{ref}}$
2. Interface conditions	$\Gamma_{\mathrm{wall}}$: v=0
	$\Gamma_{\mathrm{interface}}$: $T_{\mathrm{fluid}}=T_{\mathrm{tissue}}$
	$k_{\mathrm{fluid}}\nabla T_{\mathrm{fluid}}\cdot n=k_{\mathrm{tissue}}\nabla T_{\mathrm{tissue}}\cdot n$
	$C_{\mathrm{fluid}}=C_{\mathrm{tissue}}$
	$D_{\mathrm{fluid}}\nabla C_{\mathrm{fluid}}\cdot n=D_{\mathrm{tissue}}\nabla C_{\mathrm{tissue}}\cdot n$
3. Open heat loss	$\Gamma_{\mathrm{open}}$: $-k\nabla T\cdot n=q_{0}=600\;{W/m}^{2}$
4. Surface heat loss	$\Gamma_{\mathrm{tissue}}$: $-k\nabla T\cdot n=\frac{1}{R_{\mathrm{thermal}}}\left(T-T_{\mathrm{tissue}}\right)$
5. Drug transport to tumor	$\Gamma_{\mathrm{tumor}}$: $C=C_{\mathrm{oxali}}\left(t\right)$
	$\Gamma_{\mathrm{tumor}-\mathrm{host}}$: $C_{\mathrm{tumor}}=C_{\mathrm{host}}$
	$D_{\mathrm{tumor}}\nabla C_{\mathrm{tumor}}\cdot n=D_{\mathrm{host}}\nabla C_{\mathrm{host}}\cdot n$

**Table 2:** Organ-specific thermal properties used in the present study [36].

Organ	$w_{b}$ (mL/min/kg)	$\rho_{t}$ (kg/m^3^)	$k_{t}$ (W/m/K)	$c_{t}$ (J/kg/K)
Liver	860	1079	0.52	3540
Pancreas	767	1087	0.51	3164
Intestine	765	1088	0.54	3655
Spleen	1557	1089	0.53	3690
Kidneys	3795	1066	0.53	3763
Stomach	460	1088	0.53	3690
Skin	106	-	-	-
Peritoneal Wall	33	-	-	-

**Table 3:** Parameters applied in the drug transport model.

Parameter	Value	Reference
$L_{P}$ [m/Pa/s] (Tumor)	$2.10\times 10^{-11}$	[37]
$L_{P}$ [m/Pa/s] (Normal)	$2.70\times 10^{-12}$	[37]
S/V [m^−1^] (Tumor)	$1.00\times 10^{4},2.00\times 10^{4}$	[37]
S/V [m^−1^] (Normal)	$7.00\times 10^{3}$	[37]
$P_{c}$ [m/s] (Tumor)	$1.43\times 10^{-6}$	[38]
$P_{c}$ [m/s] (Normal)	$2.2\times 10^{-9}$	[37]
$P_{v}$ [Pa]	$2.08\times 10^{3}$	[37]
$P_{i}$ [Pa] (Tumor)	$2.67\times 10^{3}$	[26]
$P_{i}$ [Pa] (Normal)	$1.33\times 10^{2}$	[39]
$\pi_{i}$ [Pa]	$1.33\times 10^{3}$	[37]
$\pi_{v}$ [Pa]	$2.67\times 10^{3}$	[37]
*c* [-] (Tumor)	0.82	[37]
*c* [-] (Normal)	0.91	[40]
σ [-]	0.95	[37]
$T_{v}\left[\text{°}C\right]$	37	[41]
*D* [m^2^/s]	$3\times 10^{-9}$	[32]
β [s^−1^]	$7.32\times 10^{-4}$	[38]
$K_{12}$ [$h^{-1}$]	2.8	[42]
$K_{21}$ [$h^{-1}$]	1.1	[42]
$K_{e}$ [$h^{-1}$]	3.9	[42]

**Table 4:** Mean IC50 and TER of oxaliplatin based on data reported for CMS4 colorectal cancer [3].

Temperature	37 °C	38 °C	39 °C	40 °C	41 °C	42 °C	43 °C
IC50 [μM]	537.7	353.7	314.3	311.3	245.0	229.7	220.0
TER	1.0	1.43	1.6	1.63	2.07	2.13	2.23

**Table 5:** Steady-state-induced temperatures and corresponding IC50 values of oxaliplatin at abdominal organ surfaces.

Config. 1	Pancreas	Liver	Intestine	L.Kidney	R.Kidney	Stomach	Spleen
Temp. [°C]	41.3	40.4	40.1	38.4	40.7	41.7	41.7
IC50 [μM]	241	285	305	338	265	234	234
**Config. 2**							
Temp. [°C]	41.8	40.6	41.0	40.5	40.9	42.0	41.9
IC50 [μM]	232	271	245	278	251	229	231

**Table 6:** Simulation-based comparison showing equivalence of drug penetration effects between standard oxaliplatin-based HIPEC treatment and combination therapy with bevacizumab. The combination therapy achieves comparable penetration effects at a lower oxaliplatin dose (160 μM) during 30 min HIPEC procedure.

Baseline Strategy	Equivalent Combination	Effect
Oxaliplatin 200 μM	Oxaliplatin 160 μM + Bevacizumab 0.5 mg/kg	Comparable drug penetration
Oxaliplatin 300 μM	Oxaliplatin 160 μM + Bevacizumab 5 mg/kg	Comparable drug penetration

## Data Availability

The data supporting the findings of this study will be made available by the corresponding author upon reasonable request.

## Abbreviations

The following abbreviations are used in this manuscript:

AUC	Area under the curve
CFD	Computational fluid dynamics
CMS	Consensus molecular subtype
CRS	Cytoreductive surgery
HIPEC	Hyperthermic intraperitoneal chemotherapy
IPEC	Intraperitoneal chemotherapy
MVD	Microvascular density
PMCRC	Peritoneal metastasis of colorectal cancer
TER	Thermal enhancement ratio
VEGF	Vascular endothelial growth factor
